# Selected Heavy Metals Removal From Electroplating Wastewater by Purified and Polyhydroxylbutyrate Functionalized Carbon Nanotubes Adsorbents

**DOI:** 10.1038/s41598-018-37899-4

**Published:** 2019-03-14

**Authors:** Mercy Temitope Bankole, Ambali Saka Abdulkareem, Ishaq Alhassan Mohammed, Stephen Shaibu Ochigbo, Jimoh Oladejo Tijani, Oladiran Kamaldeen Abubakre, Wiets Daniel Roos

**Affiliations:** 1 Department of Chemistry, Federal University of Technology, PMB.65, Minna, Niger State Nigeria; 2 Department of Chemical Engineering, Federal University of Technology, PMB.65, Minna, Niger State Nigeria; 3 Department of Mechanical Engineering, Federal University of Technology, PMB.65, Minna, Niger State Nigeria; 4 Department of Physics, University of the Free State, P.O. Box 339, ZA-9300 Bloemfontein, Republic of South Africa; 5 Nanotechnology Research Group, Centre for Genetic Engineering and Biotechnology (CGEB), Federal University of Technology, P.M.B 65, Bosso, Minna, Niger State Nigeria

## Abstract

This research investigated the removal of heavy metals (As, Pb, Cr, Cd, Ni, Cu, Fe, and Zn) via batch adsorption process from industrial electroplating wastewater using two different nano-adsorbents; purified carbon nanotubes (P-CNTs) and polyhydroxylbutyrate functionalized carbon nanotubes (PHB-CNTs), both produced through catalytic chemical vapour deposition (CCVD) method. HRSEM, HRTEM, XRD, DLS, BET, FTIR, XPS, TGA, pH drift and Raman spectroscopy were used to characterize the developed nano-adsorbents. In the batch adsorption process, the effects of contact time, dosage, temperature and pH were studied. Both nano-adsorbents gave optimum contact time, equilibrium time, optimum dosage, and pH of 10 minutes, 70 minutes, 20 mg, and 5.63–5.65 respectively. The heavy metals removal efficiencies by the nano-adsorbents followed the order of PHB-CNTs > P-CNTs based on ion exchange and electrostatic forces mechanism. For P-CNTs and PHB-CNTs, the equilibrium sorption isotherm suits temkin model, kinetic data fitted to pseudo-second order based on the linear regression correlation coefficient, and the thermodynamic study established spontaneity and endothermic nature of the adsorption process. The findings in this research conclude that both nano-adsorbents have exceptional capacity to remove heavy metals from the adsorbate, with PHB-CNTs possessing better quality. The treated adsorbate meets the standard for industrial or irrigation re-use.

## Introduction

Water is life and needs to be preserved and protected for posterity sake. World Health Organization (WHO) has predicted that more than three billion people of the world population will not have access to safe water by 2025, and one-third of this figure lives in water-stress countries and further anticipated to increase to two-third by 2050^1^. The world today is faced with the challenges of environmental degradation particularly water pollution via industrial activities such as mining, tannery, textile, electroplating, pharmaceutical, and metallurgical, petrochemicals and others^2–4^. Other sources of water pollution include poor agricultural practices, sewage and septic tank leakage, flooding caused by global warming and climate change.

One of the trending industrial activities is plating. Plating is the application of metal to a surface of a material for protection against corrosion which is carried out via electroplating process^5^. The major element used for plating are brass, zinc, silver, gold, nickel, copper, iron, aluminum, lead, tin, platinum and chromium plating^6,7^. Industrial electroplating process involves acid pickling, alkaline cleaning, plating, and rinsing which produces wastewater in large quantities containing high level of heavy metals, chemical oxygen demand (COD), cyanides, nitrates, and sulphate complexes to mention but a few^5,7–9^.

Moreover, since 1947, purification of industrial electroplating wastewater has been seen as a major challenge^10^ due to its chemical composition, its effects on the environment without proper and legal policies guiding its release into the environment. Industrial electroplating wastewater has very high concentration of heavy metals such as iron, zinc, nickel, cadmium, chromium, lead, copper, arsenic amongst others^11–13^. The severity of electroplating wastewater pollution has led to discharge of heavy metals which are toxic, recalcitrant and non-biodegradable in the environment and exposure to human, terrestrial and aquatic organisms at high concentration can cause disruption of the endocrine system, respiratory organs, and low intelligent quotient to mention but a few. Acute exposure of some of these heavy metals to the environment and animals may cause nausea, vomiting, kidney and liver damage, irritation to the skin as well as lung cancer^14^. In addition, electroplating wastewater is highly coloured, turbid, too acidic (low pH), and can cause bio-distortion when leached into plant via soil, which indirectly causes toxins in foods and negative effects on the ecosystem.

Several conventional wastewater treatment technologies such as electrocoagulation, flocculation, co-precipitation, filteration, reverse osmosis, membrane bioreactor, electrodialysis, ultrafiltration, biosorption, solvent extraction, ion exchange, and wetland technology^4,7,12,15–17^ amongst others are not effective for the removal of heavy metals in the range of 1–100 mg/L. The reduction/removal of pollutants by conventional treatment techniques is difficult^18^ and a need for an innovative technique is desired.

Adsorption technology is mostly applied to sequester different biodegradable and non-biodegradable contaminants from wastewater^19,20^. The utilisation of carbon nanotubes as an adsorbent compared to widely used activated carbon is expected to produce satisfactory result due to its unique mechanical, electrical and thermal properties. CNTs are entangled tubes with aggregated pores that adhere to each other based on van der Waals forces of attraction^21^. Additionally, CNTs has outstanding surface area, pore volume and pore size^22^ and adsorption usually occurred at the external surface of the nanotubes due to its closed ends nature compared to activated carbon where the interior space were used for adsorption process^23–25^. The external surfaces of CNTs have potential sites and pores that can be used for adsorption of metallic ions^21^.

On the other hand, the as-prepared CNTs in the form of fullerenes, carbon nanoparticles and amorphous carbon usually contain impurities which affect its performance and such impurities from CNTs can be removed by three different methods namely; chemical, physical and multi-step purification^26^. Chemical purification method entails selective oxidation of carbonaceous impurities especially residual metallic constituents by acid treatment^27^. The physical purification method separates CNTs from impurities based on the differences in their physical properties; size, gravity, magnetic properties, amongst others. The multi-step purification is a combination of two earlier stated purification techniques that give rise to high-quality CNTs with increased yield^28^.

Furthermore, functionalization or modification of CNTs is one of the distinctive ways of enhancing the adsorptive surface properties of CNTs based on the attached functional groups by covalent or non-covalent bonding onto the side wall or tip of CNTs^29^. This will not only improve the hydrophilicity and solubility behaviour of CNTs in water but equally provides better interaction with the adsorbate compared to hydrophobic CNTs^30^. The enrichment of CNTs via functionalization with a polymer has resulted in higher electric conductivity and mechanical properties compared to acid treated purified CNTs^3,31–33^. Polymer functionalized CNTs have high adsorptive capacity than ordinary CNTs due to increase in functional groups attachments, surface area, porosity and crystallinity. In this study, the purified CNTs were functionalized with a non-toxic Polyhydroxylbutyrate (PHB). The polymer is not soluble in water however dissolve in chloroform and chlorinated hydrocarbons and showed greater resistance to hydrolytic degradation unlike other biodegradable polymers^34^. PHB is highly permeable to oxygen, biocompatible for medical applications but cannot be decomposed by ultra-violet radiation^35^. PHB also sinks in water thereby facilitating its anaerobic biodegradation. Its stickiness decreases during melting thus enhancing the material as an adsorbent. Composites of PHB with CNTs showed a better solubility in PHB/chloroform solutions and resulted in higher electric conductivity and higher mechanical properties compared to purified CNT alone^36^. In view of the aforementioned, this study investigated the adsorptive behaviour of multi-step purified carbon nanotubes and its polyhydroxylbutyrate functionalized counter-part for removal of selected heavy metals from industrial electroplating wastewater.

## Results and Discussion

### Characterization of the nano-adsorbents

The surface area, attached functional groups, and morphology of adsorbents are considered necessary for an effective adsorption process. In this study the nano-adsorbents were characterized to be able to ascertain their adsorptive properties and capacity for the specific usage.

**Figure 1:**
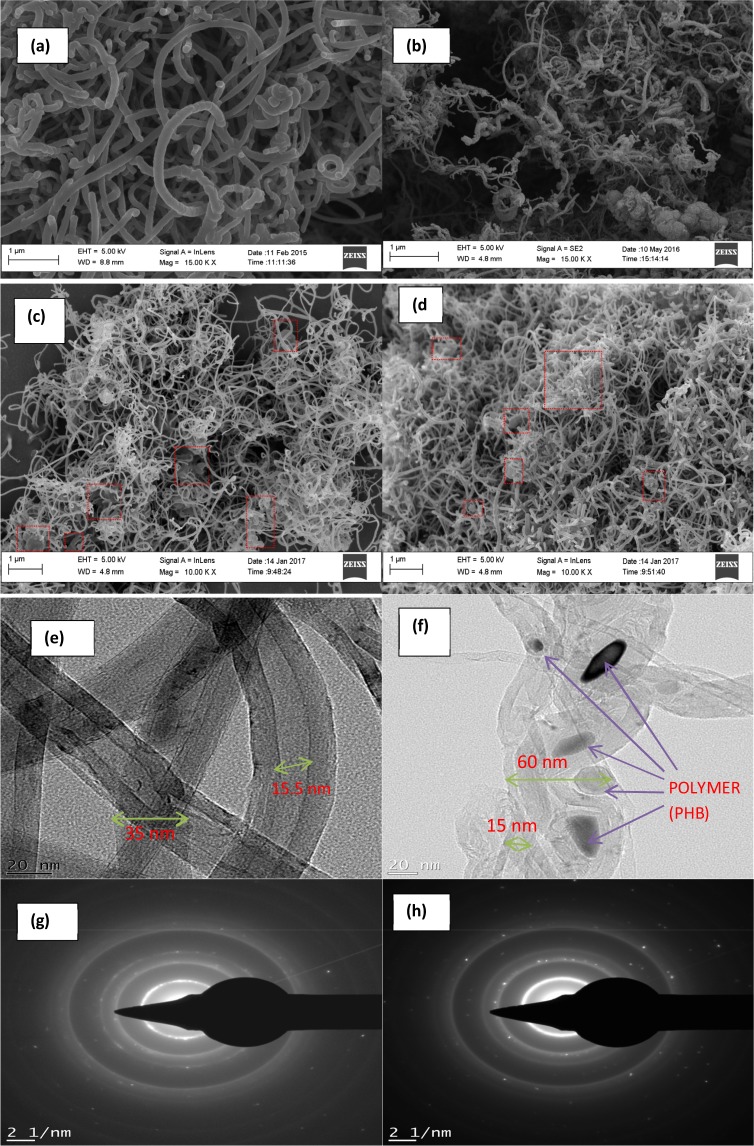
High resolution scanning electron microscope images of the (**a**) P-CNTs and (**b**) PHB-CNTs (**c**) P-CNTs utilized for electroplating wastewater treatment and (**d**) PHB-CNTs utilized for electroplating wastewater treatment. HRTEM images of the (**e**) P-CNTs and (**f**) PHB-CNTs. SAED patterns of (**g**) P-CNTs and (**h**) PHB-CNTs.

High resolution scanning electron microscope (HRSEM) micrograph in Fig. 1(a) for P-CNTs revealed the formation of uniformly distributed smooth tubular structures which support the findings of Lu and Su^37^. Also, in the case of polyhydroxylbutyrate functionalized CNTs (see Fig. 1(b)), the HRSEM confirmed the presence of rough and thick aggregated morphologies on the CNTs outer surface similar to polymerized beehive like structure^38^. The rough clustering was dominant in PHB-CNTs, due to its higher molecular weight and non-solubility of PHB in water. In the same vein, it was found that even after batch adsorption treatment of the wastewater with the two developed nano-adsorbents, the curved tubular structures of the P-CNTs observed in Fig. 1(a) were still retained except with interference and adherence of flakes of some heavy metals as indicated in the red dotted rectangular-boxes in Fig. 1(c). Conversely, rough agglomerated clusters of PHB-CNTs earlier observed in Fig. 1(b), disappeared without destroying the tubular network of CNTs after application during the batch adsorption process (see Fig. 1(d)). This shows that functionalization using polyhydroxybutyrate did not destroy the CNTs morphologies. In addition, the agglomerated surface of PHB-CNTs reduces and literally disappears during the interaction between the adsorbate and the adsorbent because the heavy metals in the electroplating wastewater are being removed to the surface of the adsorbent (PHB-CNTs). However, there were slight breakages, cuts and shortenings of the nanotubes of the used nano-adsorbents, as a result of attachment of the pollutants to the surface. This is due to reduction in the inter-molecular forces within the isolated CNTs possibly due to adsorption of pollutants onto the surface of the outer-wall of the aggregated CNTs structure. Cumulatively, the HRSEM results Fig. 1(a–d) implies that the nano-adsorbents morphology is retained, thereby could be recycled for subsequent application.

Figure 1(e,f) depict the HRTEM micrographs of the prepared nano-adsorbents. According to Fig. 1(e), graphitic multi-wall carbon nanotubes structure (MWCNTs) with inner diameter of 15.5 nm and defective etch on the wall of the CNTs was noticed in P-CNTs, while that of PHB-CNTs reduced to 15 nm, due to pressure pull caused by polymer infringement on the side-wall of the nanotube. However, the inner-outer diameter increased after functionalization because of increase in the layer on the outer wall of nanotube as a result of polymer placement; P-CNTs (35 nm) and PHB-CNTs (60 nm). Specifically, it is obvious that the polyhdroxylbutyrate was agglomerated and contain thick black cluster encapsulated within lattice fringes as indicated by the arrows (see Fig. 1(f)). The result of Energy Dispersive X-ray spectroscopy (EDS) analysis of the nano-adsorbents in Table 1 revealed the presence of carbon as the dominant element in both nano-adsorbents. Other elements such as calcium, cobalt and iron originated from the bimetallic catalyst used for CNTs growth. However, these elements were trapped within the wall of the CNTs while the residual sulphur detected was ascribed to H_2_SO_4_ and SOCl_2_ used during purification and functionalization of the nano-adsorbents. As shown in Table 1, a slight increase in atomic percentage of carbon content from 93% in P-CNTs to 93.4% (PHB-CNTs) was observed and the additional increment was contributed by PHB.

**Table 1:** EDS Results of P-CNTs and PHB-CNTs.

Element	P-CNTs (%)	PHB-CNTs (%)
C	93.00	93.4
O	4.13	4.3
Ca	0.06	—
Fe	0.80	0.74
Co	0.57	0.57
S	1.44	0.99
	**100**	**100**

The degree of crystallinity of the nano-adsorbent is a contributing factor for an effective adsorption and this was examined using Selected area electron diffraction (SAED) and the results are presented in Fig. 1(g,h). The bright spots and rings of the SAED patterns suggest that the two materials are polycrystalline in nature and each ring depicts diffraction pattern of crystals of similar size with each bright spots reflecting individual peaks. The SAED patterns of both samples further confirmed the graphitic nature of MWCNTs due to the presence of the bright innermost ring assigned to reflection plane (002) of a typical graphitic carbon. As shown in Fig. 1(g), the P-CNTs rings observed at distances from zero (reciprocal lattice spacing (*1/d*)) were given as 2.8, 4.4, 4.8 and 8 nm^−1^ which closely agreed with the values reported for graphite. Similar trend was observed in PHB-CNTs (see Fig. 1(h)) rings with *1/d* of 2.7, 4.6, 6.6, and 8.6 nm^−1^ which is also in conformity with SAED patterns of MWCNTs and brighter spots reflecting higher degree of crystallinity, as reported by Bankole *et al*.^39^ for purified and polyethylene glycol modified carbon nanotubes.

The particle size of carbon nanotubes was determined by dynamic light scattering (DLS) technique. Not only that, the Z-average, dispersity index, length, diameter and aspect ratio of the purified and PHB functionalized MWCNTs were analyzed by DLS (Nanozeta-sizer) in conjunction with HRTEM and the results obtained are shown in Fig. 1(e,f) (diameter) and Table 2 respectively. The diameter of each nano-adsorbent was derived from the HRTEM results, and used to estimate the length and aspect ratio of the samples. For polydispersive index *Đ* = 0, the sample is monodispersed with uniform size and shape, when *Đ* is the range of 0.1–0.4, the sample is moderately polydispersed while samples with *Đ* > 0.4 is broadly polydispersed^40^. In this work, it was noticed that both samples were broadly polydispersed however the degree of dispersity of PHB-CNTs is greater than P-CNTs. Aspect ratio is an important factor that determines the applicability of CNTs in various areas, and as reported by Abu Al-Rub *et al*.^41^, the aspect ratio of CNTs produced by CCVD process ranged from 157 to 3,750. For the nano-adsorbents, P-CNTs have the highest aspect ratio of 3200 compared to PHB-CNTs with aspect ratio of 400. Kim *et al*.^42^ reported that the aspect ratio of CNT is directly proportional to Young’s modulus, an essential property for the development of nano-adsorbent during the adsorption process.

**Table 2:** DLS (Nanozeta-sizer) of P-CNTs and PHB-CNTs.

Nano-Adsorbent	Z-Average (D_h_)	Polydispersity (*Đ*) Index	Length (µm)	Diameter (nm)	Aspect Ratio
P-CNTs	832.1	0.410	4.20	35	3200
PHB-CNTs	20350	0.908	167.67	60	400

The surface area of the nano-adsorbents was determined by Brunauer-Emmett-Teller (BET) N_2_ adsorption-desorption analysis and the results presented in Table 3. The relative pressures (*P*/*P*o) of P-CNTs and PHB-CNTs are 5.84 × 10^−2^ and 0.307 respectively, indicating type II of a typical mesoporous material. Upon functionalization of the P-CNTs with PHB, an increase in the surface area and porosity of PHB-CNTs was observed (see Fig. 1b). The increment in the surface area of PHB-CNTs may be attributed to low stickiness and high rate of oxygen penetration of PHB on CNTs^43^ (see Table 3). The porosity of the nano-adsorbents was also considered; a passage between the external and internal surface of the the nano-adsorbent that permit free flow of gases and vapour. The pore radius of P-CNTs and PHB-CNTs are 3.156 nm and 3.293 nm respectively. The pore radii of both nano-adsorbents fall within 2–50 nm, and are classified as mesoporous materials according to IUPAC classifications^43^. This implies that the nano-adsorbents will act as passages or transport system to the micropores, hereby resulting to condensation of the capillary with the adsorption/desorption isotherm by multilayer formation.

**Table 3:** BET Analysis of P-CNTs and PHB-CNTs.

Nano-Adsorbent	Specific Surface Area (m^2^/g)	Total Surface Area (m^2^)	Pore Volume (cm^3^/g)	Pore Radius (nm)
P-CNTs	227.637	100.16	0.0827	3.156
PHB-CNTs	253.189	111.40	0.0876	3.293

**Figure 2:**
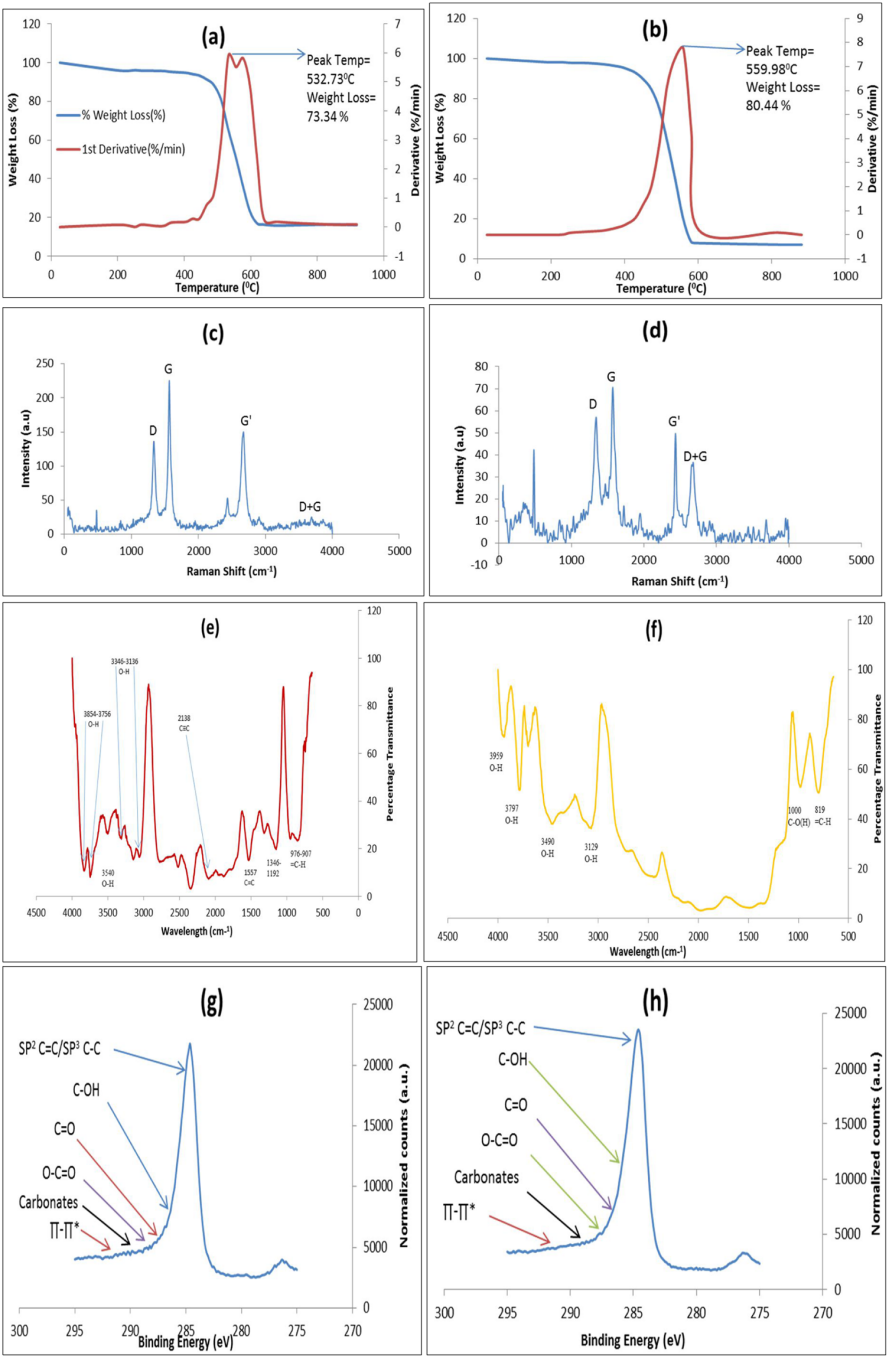
TGA curve of (**a**) P-CNTs and (**b**) PHB-CNTs obtained at 10 °C/min in N_2._ Raman spectrum of (**c**) P-CNTs and (**d**) PHB-CNTs. FTIR spectrum of the (**e**) P-CNTs and (**f**) PHB-CNTs. XPS C 1 s of the (**g**) P-CNTs and (**h**) PHB-CNTs.

Thermogravimetric analysis (TGA) was used to investigate the thermal stability and purity of CNTs. Figure 2(a,b) shows the TGA plots of the two nano-adsorbents heated in N_2_ atmosphere at a heating rate of 10 °C/min. The TGA profile of P-CNTs in Fig. 2(a) indicates that the material degraded fast in the temperature range of 165–445 °C possibly due to residual Fe-Co/CaCO_3_ catalyst after the CNTs synthesis in CVD reactor. However, after purification, the degradation temperature of purified carbon nanotubes was enhanced to 532.73 °C with 73.34% weight loss. The residual material after the decomposition of the P-CNTs was 26.66% (carbonaceous material), which is a typical profile for MWCNTs^44^. Also the corresponding DTG graph shows that the P-CNTs sample started burning at ∼165–445 °C (Fig. 2(a)). The possible reason for this low ignition temperature may be ascribed to the presence of residual catalyst metal particles (Fe-Co) derived from the CCVD synthesis process. The double peaks in Fig. 2(a) may be attributed to the oxidation of CNTs and residual impurities from Fe-Co/CaCO_3_. However, these two peaks disappeared upon polymer functionalization into a single peak (Fig. 2(b)); this means that the polymers used for functionalization suppressed the residual impurities. In Fig. 2(b), the functionalized PHB-CNTs revealed a peak degradation temperature at 559.98 °C with weight loss of 80.44% leaving the residual material at 19.56%. Consequently, the most thermally stable with least residual material among the nano-adsorbents is PHB-CNTs.

Raman spectroscopy analysis was used to characterize the purified and polymer functionalized carbon nanotubes^45^. The Raman spectrum of the purified carbon nanotubes display in Fig. 2(c) indicates a shift and a peak at 479.51 cm^−1^, corresponding to the deposit of the metal catalyst entrapped within the wall of the CNTs. Also, two sharp peaks were noticed at 1329 cm^−1^ and 1563 cm^−1^ correspond to the disorder induced band (*D* band) and the Raman allowed tangential mode (*G* band) respectively. The Raman spectrum also exhibited a band at 2330, 2425, 2901, 2958 cm^–1^ called second-order harmonic band *G*’ (Graphite) band, attributed to the overtone of the *D* band. The *D* mode indicates the disordered features of the carbon and defects in the curved graphite sheet, *sp*^3^ carbon with other impurities while the *G* mode is associated with the ordered crystalline graphite in the CNTs, in accordance with the work of Zdrojek *et al*.^46^. The Raman spectrum shows a second-order harmonic band *G*’ (Graphite) at 2426 cm^−1^ and 2664 cm^−1^, and a peak at 3638 cm^−1^ corresponding to the *D* + *G* band^47^. The polymer functionalized carbon nanotubes is represented in Fig. 2(d) with a peak at 479.51 cm^−1^, attributed to impurities as in the case of purified CNTs (Fig. 2(c)). In contrast to P-CNTs, there were sharp peaks at 1349 cm^−1^ (*D* band), 1564 cm^−1^ (G band), 2654 cm^−1^ (*G’* band) and 3677 cm^−1^ (*D* + *G* band). The results showed that there is a variation in the quality of the nano-adsorbents due to existence of variable intensities of the *D*-band and *G*-band respectively. The intensity ratio of the *D* band to *G* band (*I*_*D*_*/I*_*G*_) which gives an estimated value of the extent of structural defects was found to be ~0.56 and ~1.28 for the P-CNTs and PHB-CNTs respectively. This is an indication of disparities in the structures of the P-CNTs and PHB-CNTs samples which further corroborated HRSEM (Fig. 1(a,b)) and HRTEM (Fig. 1(e,f)) results.

The FTIR spectra for P-CNTs and PHB-CNTs is given in Fig. 2(e,f). Figure 2(e) for P-CNTs revealed the occurrence of a stretching vibrations band corresponding to the -OH groups in the following ranges: 3346 to 3136 cm^−1^, 3540 cm^−1^and 3854 to 3756 cm^−1^. While the vibration bands of carbonaceous species such as C≡C occurred at 2138 cm^−1^, C=C at 1557 cm^−1^, C-O bonds of the carboxyl group at 1346 to 1192 cm^−1^, and =C-H bond at 976 to 907 cm^−1^. Figure 2(f) depicted the spectrum of PHB-CNTs with the presence of stretching vibrations for -OH groups at 3959 and 3797 cm^−1^ (sharp peaks), 3490 and 3129 cm^−1^ (broad peaks), C–O(H) bonds of the carboxyl group at 1000 cm^−1^ (sharp peak) and =C-H bond at 819 cm^−1^. The PHB-CNTs clearly showed peaks of different functional groups and the degree of functionalization was related to the hydrophilicity of the CNTs which affects the adsorption process.

High resolution X-ray Photo-elecron Spectroscopy (XPS) spectrum of adventitious C (1 s) peak of P-CNTs and PHB-CNTs is shown in Fig. 2(g,h). The structure (graphite) of P-CNTs and PHB-CNTs was revealed through C (1 s) peak at the binding energies of 284.8 eV and 284.65 eV respectively. In addition, other peaks between the binding energies of 287.7–290.8 eV (see Fig. 2(g)) and 287.55–290.65 eV (see Fig. 2(h)) were ascribed to carboxyl and carbonyl groups attached to the surface of the nano-adsorbents during purification and functionalization of the CNTs. While the remaining compliment peaks in both Fig. 2(g,h) are categorised to as transition loss peaks (π-π*), connoting the attractive chemical force on the surface of CNTs. These functional attachments will invariably increase the sequestration ability of the nano-adsorbents.

### Electroplating Wastewater Treatments

According to Table 4, the level of Cr concentration was 72.342 mg/L and reduced drastically after treatment and falls within the range of 0.136–0.9005 mg/L. The discharge limit of wastewater containing Cr (VI) into the environment (surface waters) is regulated at <0.05 mg/L and 0.1 mg/L to inland surface waters according to the WHO and EPA^48,49^, whereas the total chromium discharged is regulated to be <2 mg/L. From Table 4, it was observed that the amount of Cd in the electroplating wastewater is 3.02 mg/L and after treatment with the nano-adsorbents via batch adsorption process, the value falls within the range of 0.02–0.04 mg/L. The allowable limit of Cd in drinking water according to NIS^50^, WHO^48^ and EPA^49^ is <0.003 mg/L and <0.005 mg/L respectively. Both P-CNTs and PHB-CNTs were able to sequester 99% Cd from the electroplating wastewater.

**Table 4:** The mean concentration of heavy metals in electroplating wastewater (before and after adsorption process).

Physico-chemical parameters	Raw value	Batch adsorption by P-CNTS	Batch adsorption by PHB-CNTS	WHO/EPA Permissible limits	NIS Permissible limits
pH	0.83	5.63	5.65	5.5–8.5	6.5–8.5
Iron (mg/L)	127.5	18.01	23.115	0.3/0.2	0.3
Nickel (mg/L)	106.1	8.2325	10.1105	0.07/0.02	0.02
Cadmium (mg/L)	3.02	0.04	0.02	0.005	0.003
Lead (mg/L)	4.94	0.0160	0.0395	0.01/0.05	0.01
Copper (mg/L)	97.57	5.8575	6.4735	2/0.05	1
Zinc (mg/L)	167.6	10.09	8.215	5	3
Chromium (mg/L)	72.34	0.1360	0.9005	0.05	0.05
Arsenic (mg/L)	58.03	0	0	0.01/0.05	0.01

Key: World Health Organization^48^, Environmental Protection Agency^49^ and Nigerian Industrial Standard^50^.

Also in Table 4, it was noticed that the Cu concentration in the wastewater is 97.57 mg/L and reduced to within the range 5.8575–6.4735 mg/L corresponding to 94% removal after treatment. According to Table 4, the Ni content before treatment was 106.105 mg/L, and after treatment under the applied experimental conditions fall within the range of 8.232–10.1105 mg/L (approximately 92% removal). In the same vein, the concentration of Fe in the electroplating wastewater prior to treatment was 127.53 mg/L and decreased to within the range 18.01–23.115 mg/L (reduction of approximately 85%) after treatment.

Zn concentration before treatments was 167.625 mg/L and after adsorption with the nano-adsorbents falls within 8.215–10.09 mg/L (approximately 95% removal). While in the same Table 4, the concentration of As in the electroplating wastewater was 58.039 mg/L and after treatment to be 0 mg/L for both nano-adsorbents. This result fit with the standard permissible limit. In Table 4, Pb concentration before treatment was 4.94 mg/L and after treatment was in the range of 0.0160–0.0395 mg/L. It can be inferred from the results obtained that the nano-adsorbents were suitable for the treatment of electroplating wastewater under the applied experimental conditions.

### Effects of contact time on heavy metals removal from electroplating wastewater

**Figure 3:**
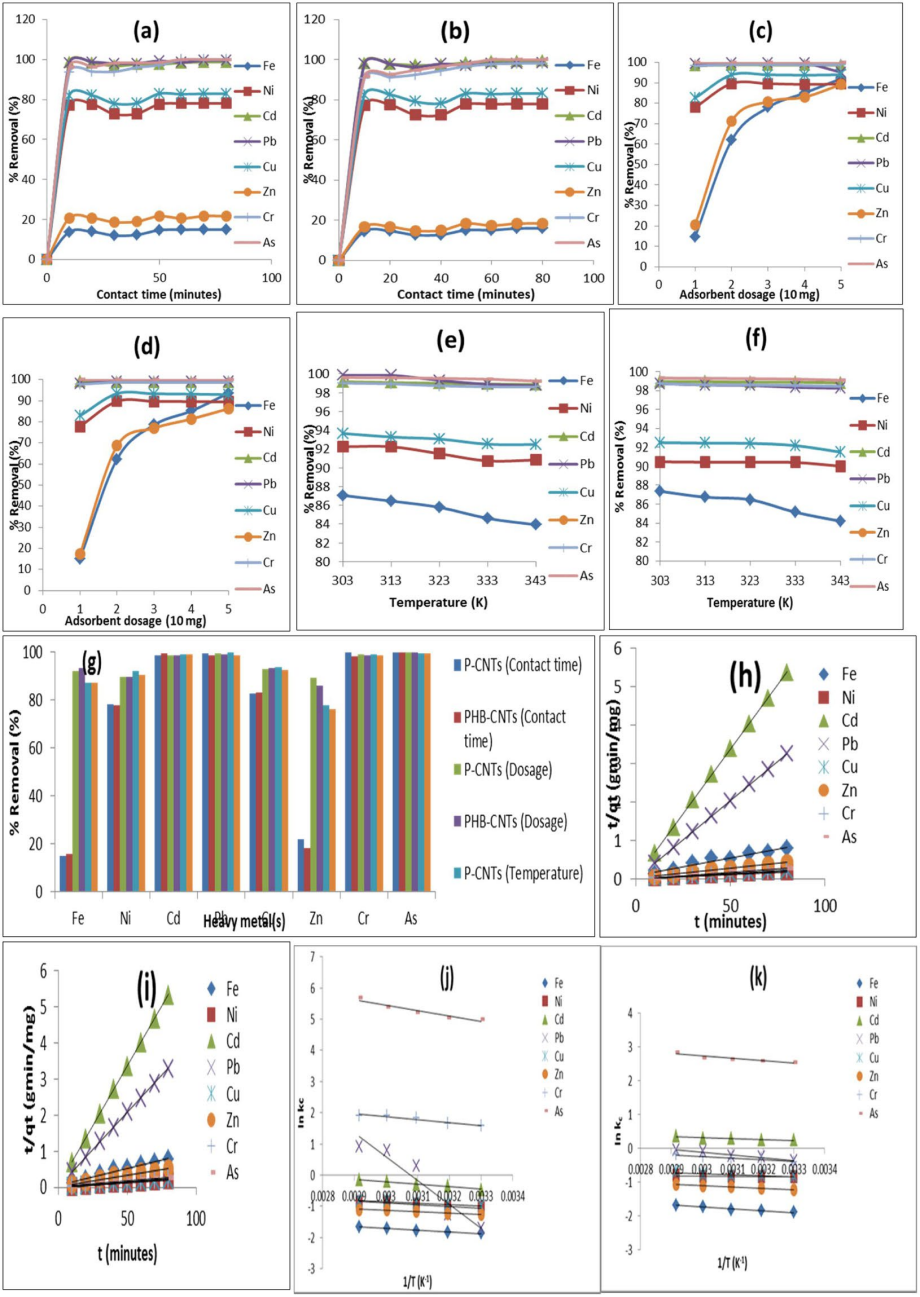
Effects of contact time on heavy metals removal onto (**a**) P-CNTs and (**b**) PHB-CNTs. Effects of adsorbent dosage on heavy metals removal onto (**c**) P-CNTs and (**d**) PHB-CNTs. Effects of temperature on heavy metals removal onto (**e**) P-CNTs and (**f**) PHB-CNTs. (**g**) Comparative effects of the adsorption/removal factors of heavy metals from electroplating wastewater. (**h**) Pseudo second order rate equation plot for heavy metals removal onto P-CNTs. (**i**) Pseudo second order rate equation plot for heavy metals removal onto PHB-CNTs. (**j**) Vant’ Hoff Plot of heavy metals adsorption using P-CNTs. (**k**) Vant’ Hoff Plot of heavy metals adsorption using PHB-CNTs.

The influence of contact time on the selected heavy metals uptake using the two nano-adsorbents is illustrated in Fig. 3(a,b). It was noticed that both P-CNTs and PHB-CNTs shows similar removal patterns under the applied conditions. It can be seen that increase in contact time correspond to increase in percentage removal efficiency of the selected heavy metals irrespective of the nano-adsorbents. It is worthy to note that, there is no significant improvement in the percentage metals removed after 10 minutes for both nano-adsorbents. Thus, the optimum contact time to achieve maximum removal of the metal ions was 10 minutes. This implies that the adsorption sites were saturated due to strong competition among the selected heavy metals and further increase in contact time did not lead to any meaningful percentage metal removal suggesting the occurrence of biphasic kinetics mechanism. The fast phase which was an instant sorption occurred within the first 10 minutes for some metals due to availability of binding sites of the nano-adsorbents, thus indicating that phase as fast phase. The smaller and slower removal of heavy metals in the wastewater by the nano-adsorbents accounted for the second phase and at contact time of 70 minutes, equilibrium was attained. At equilibrium time, there was maximum uptake capacity of the heavy metal ions due to saturation of the adsorption sites. After the equilibrium time the removal rate became constant. This occurrence could be as a result of inaccessibility of the unoccupied binding sites due to repulsive forces between the heavy metals and the nano-adsorbents. The observed biphasic mechanism in this study is similar to the finding of Wu *et al*.^51^ who had earlier observed that adsorption mechanism involved external and internal diffusion processes. It was noticed that P-CNTs and PHB-CNTs successfully removed 15.11% and 15.92% for Fe, 78.06% and 77.95% for Ni, 98.68% and 99.34% for Cd, 99.44% and 98.85% for Pb, 82.91% and 83.08% for Cu, 21.80% and 18.34% for Zn, 99.80% and 98.19% for Cr, and 99.99% and 99.95% for As, respectively. Very importantly, the maximum percentage removal of heavy metals varied and depend on the type of nano-adsorbent. Notwithstanding, PHB-CNTs removed more of the selected heavy metals than P-CNTs under the same applied conditions due to the differences in the surface area and functionality of the nano-adsorbents.

The two nano-adsorbents have greater affinity for Cd, Pb, Cu, Cr, As more than Fe, Zn and Ni possibly due to higher concentrations of the later in the electroplating wastewater. The high abundance of Fe, Zn and Ni may be responsible for their low removal from the wastewater sample. The order of concentrations of these heavy metals in the electroplating wastewater are Cd < Pb < As < Cr < Cu < Ni < Fe < Zn. Another possible reason for the differences in the metal ions removed may be due to the nature of the ions in the aqueous media, ions with smaller sizes have been known to be heavily hydrated and become larger and bulkier than the less hydrated. This enhanced their chances of being attracted to the adsorption sites faster than the heavily hydrated ions that migrate slowly in aqueous solutions^52^. The diffusion and eventual removal of the selected heavy metals by the nano-adsorbents could also be explained according to ionic/atomic radii of these metals. Element with smaller ionic radius would diffuse faster onto the pores of the nano-adsorbents than element with larger ionic radius^53^. The order of removal of the selected heavy metals irrespective of the nano-adsorbents are as follows: As > Cr > Cd > Pb > Ni > Cu > Zn > Fe. It is also possible that the heavily hydrated ions blocked the small size ions from reaching the binding sites, thus responsible for the low removal efficiency of ions with smaller ionic radii. These reasons might be responsible for the faster removal rate of As by the nano-adsorbents than Cr, Cd, Pb, Cu, Ni, Zn and lastly Fe. The adsorption potentials of each nano-adsorbent with respect to the metal ions differ and do not follow a specific trend. These imply that the removal efficiency of the metals is nano-adsorbent specific.

### Influence of adsorbent dosage on selected heavy metals removal from electroplating wastewater

Adsorbent dosage showed a significant effect on the removal of selected heavy metals from electroplating wastewater as observed after 10 minutes (see Fig. 3(c,d)). With exception of Fe and Zn, the percentage removal of other heavy metals increases with increasing dosage of the nano-adsorbents dosage and reached a saturated point after the addition of 20 mg. This increment may be attributed to the availability of the adsorption sites which is in agreement with the findings of Gin *et al*.^54^.

The results as presented in Fig. 3(c,d) also indicated that P-CNTs and PHB-CNTs exhibited percentage removal of 92.09% and 93.56% for Fe, 89.71% and 89.70% for Ni, 98.78% and 98.82% for Cd, 99.68% and 99.20% for Pb, 93.00% and 93.37% for Cu, 89.26% and 86.21% for Zn, 99.00% and 98.75% for Cr, and 100% and 100% for As, respectively.

### Effects of pH of the nano-adsorbents on heavy metal removal from electroplating wastewater

Adsorption depends on the nature of the metal ions at the different pH values of adsorbents. The surface of an adsorbent can be neutral, positively or negatively charged; a factor of the surface functional groups. pH_zpc_ is the pH at which the adsorbent surface charge density is zero (neutral/point zero charge)^55^. The surface of the adsorbent is positively charged at a pH < pH_zpc_, and negatively charged at pH > pH_zpc_. From Table 1, the initial pH of the wastewater was 0.83 (highly acidic), and at post-treatment with the nano-adsorbents there was a shift in the pH value. The pH values of the P-CNTs and PHB-CNTs treated wastewater is 5.63 and 5.65 respectively as presented in Table 1, where adsorption was at equilibrium and maximum. The pH_zpc_ of P-CNTs is 6.2 and that of PHB-CNTs is 6.8, this shows that at pH values lower than pH_zpc_ of P-CNTs and PHB-CNTs the surface of the nano-asorbents were positively charged. This invariably led to maximum adsorption below pH_zpc_ of both nano-adsorbents as evident in the results presented in Table 1. This is because at a pH below the pH_zpc_ of the nano-adsorbents, there was maximum attraction between the anions of the heavy metals and the positive charged surface of the nano-adsorbents. However, the adsorption decreased at pH values higher than pH_zpc_ of the nano-adsorbent due to electrostatic repulsion between the anions of the heavy metals and the negatively charged surface of the nano-adsorbents.

### Effects of temperature on heavy metal removal from electroplating wastewater

The removal efficiency with respect to the applied temperature are shown in Fig. 3(e,f). It was noticed that the removal rate of all metal ions decreases with increasing solution temperature. This means there is an inverse relationship between the solution temperature and the percentage heavy metals removed an evidence of a decrease in the distribution coefficient of the metal ions between the outer and inner pores of the nano-adsorbent molecules. As a consequence, the swelling behaviour of the nano-adsorbents in the solution decreases as temperature increases. The sequence of heavy metals sorption rate by the nano-adsorbents is: P-CNTs > PHB-CNTs, with P-CNTs and PHB-CNTs removed 87.05% and 87.37% of Fe, 92.24% and 90.47% of Ni, 99.17% and 98.94% of Cd, 99.91% and 98.75% of Pb, 93.66% and 92.50% of Cu, 77.92% and 76.30% of Zn, 99.02% and 98.76% of Cr, and 99.61% and 99.32% of As, respectively. The findings of this study closely agree with Malana *et al*.^56^ who used polymeric gel as an adsorbent. The P-CNTs adsorbed heavy metals better compared to the PHB-CNTs, which could be as a result of the higher aspect ratio (see Table 2) of the former than the latter that influenced the adsorption process. Likewise, the thermal degradation of the polymer in PHB-CNTs also influenced the adsorption process. This agrees with the report of Garg *et al*.^57^, who studied the adsorption capacity of corncob based activated carbon for the removal of Cr (VI) and COD from electroplating wastewater and observed decrease in the percentage removal despite increasing the temperature (adsorption process gave maximum percentage removal at 298 K).

### Adsorption Isotherm Model

In this work, Langmuir, Freundlich, Temkin and Dubinin-Radukevich (D-R) isotherms were applied to describe the relationship between the adsorbate in the liquid phase and the adsorbate on the surface of the adsorbent. The data obtained were used to establish which of the isotherm model best described the adsorption phenomena. The regression correlation coefficient (*R*^2^) for the Langmuir, Freundlich, and D-R isotherm models were relatively small compared to that obtained for Temkin isotherm model. Generally, based on the numerical value of the correlation coefficient (*R*^2^) shown in Tables 5–6, it can be concluded that the equilibrium sorption data better described using the Temkin isotherm model. The order of fitness of the isotherm models are Temkin > D-R > Freundlich > Langmuir. The Temkin isotherm parameters for the heavy metals are presented in Tables 5 and 6. The Temkin constant (*b*_*T*_) is related to heat of sorption for the heavy metals on both nano-adsorbents. The values of (*b*_*T*_) in this study indicates a good interaction between sorbate and sorbent, which is an evidence of an ion-exchange mechanism during the adsorption process^45^.

**Table 5:** Isotherm parameters on the adsorption of the selected Heavy Metals by P-CNTs.

Isotherm models	Fe	Ni	Cd	Pb	Cu	Zn	Cr	As
**Langmuir**								
a_L_	312.50	10.09	66.67	−625	21.65	28.90	98.04	−50000
b_L_	2.31	−0.02	−11.06	−117.25	−0.004	0.1214	−0.39	−605
Q_0_	135.14	−625	−6.03	5.33	−5000	238.10	−250	82.65
R_L_	0.0034	−1.40	−0.03	−0.002	1.7313	0.0468	−0.03	−0.002
R^2^	0.0027	0.4155	0.1501	0.0074	0.4606	0.2226	0.2494	0.0074
**Freundlich**								
K_f_	160.33	5.10	189103	3.66	23.12	154.10	205.35	87.68
1/n_f_	−0.045	1.388	3.257	−0.277	1.008	0.068	2.223	−0.200
n_f_	−22.37	0.72	0.31	−3.61	0.99	14.77	0.45	−5.01
R^2^	0.0197	0.7078	0.3710	0.2291	0.6514	0.0350	0.5005	0.7094
**Temkin**								
B_T_	−2.06	345.86	31.00	−2.18	241.96	12.92	480.31	−32.89
b_T_	−1101	6.56	73.23	−1040	9.38	175.70	4.73	−69.00
A_T_	3 × 10^−32^	0.1401	30.3994	0.1759	0.3109	181441	1.6833	0.0590
R^2^	0.0021	0.7904	0.5838	0.0930	0.8191	0.0268	0.7065	0.8767
**D-R**								
K_ads_	−4 × 10^−6^	−3 × 10^−5^	−1 × 10^−7^	9 × 10^−9^	−1 × 10^−5^	−3 × 10^−5^	−5 × 10^−7^	6 × 10^−9^
q_s_	145.27	536.20	1398.42	5.15	467.41	230.81	798.23	87.68
R^2^	0.0678	0.5827	0.3581	0.181	0.6392	0.3176	0.4434	0.7094

**Table 6:** Isotherm parameters on the adsorption of the selected Heavy Metals by PHB-CNTs.

Isotherm models	Fe	Ni	Cd	Pb	Cu	Zn	Cr	As
**Langmuir**								
a_L_	227.27	10.16	−30.12	196.08	19.61	49.26	74.63	−2500
b_L_	1.6364	−0.0163	−34.6536	3.9216	−0.0012	0.2463	−0.366	−30.25
Q_0_	138.89	−625.00	0.86	50	−16667	200	−204.1	82.65
R_L_	0.0048	−1.38	−0.01	0.0491	1.1297	0.0237	−0.04	−0.0001
R^2^	0.0086	0.4319	0.8692	0.2212	0.3677	0.0341	0.4111	0.4998
**Freundlich**								
K_f_	154.24	5.44	1.4 × 10^−9^	328.78	16.80	198.79	128.07	87.68
1/n_f_	−0.313	1.365	−6.611	1.204	1.112	−0.018	1.992	−0.384
n_f_	−31.949	0.732	−0.151	0.831	0.899	−56.18	0.502	−2.602
R^2^	0.0114	0.6309	0.8697	0.5066	0.5978	0.0017	0.6460	0.7091
**Temkin**								
B_T_	−0.6750	338.89	−52.375	17.651	271.26	−1.0382	410.72	−63.26
b_T_	−3362.6	6.70	−43.34	128.59	8.37	−2186	5.53	−35.88
A_T_	2 × 10^−94^	0.1424	25.0198	36.0660	0.2578	7 × 10^−83^	1.4366	0.2295
R^2^	0.0003	0.8037	0.9683	0.7095	0.7806	0.0001	0.8031	0.8765
**D-R**								
K_ads_	−3 × 10^−6^	−3 × 10^−5^	−2 × 10^−7^	−4 × 10^−8^	−1 × 10^−5^	−3 × 10^−5^	−7 × 10^−7^	2 × 10^−9^
q_s_	145.71	542.62	6.5 × 10^−5^	71.25	445.50	210.76	755.21	87.68
R^2^	0.0730	0.6103	0.8930	0.4741	0.5278	0.1424	0.6210	0.7091

### Adsorption Kinetic Model

The dynamic and mechanism of adsorption of the selected heavy metals on the nano-adsorbents was explained by four kinetic models (Pseudo-first order, Pseudo-second order, Elovich and Fractional power kinetic model). In this study, the model with highest value of correlation coefficients (*R*^2^) was chosen as the better fitted and it was found that out of the four models used only pseudo-second order kinetic model with *R*^2^ (≥0.9563) better described the adsorption kinetics than others (Fig. 3(h,i)). This showed that the removal of the selected heavy metals from electroplating wastewater was dependent on the concentration of the heavy metals and the dosage of the nano-adsorbents. The order of fitness of pseudo second order kinetics by the nano-adsorbents according to (*R*^2^) value is as follows: PHB-CNTs (*R*^2^ = 0.9937) > P-CNTs (*R*^2^ = 0.9928), which can be linked to the differential potential of the material to hold water. More so, the initial adsorption rate (*h* (mg/g/min) as presented in Tables 9 and 10 (supplementary file), showed that for P-CNTs, the order of increasing *h*-value is Cu > As > Ni > Cr > Pb > Zn > Cd > Fe, while for PHB-CNTs, the order is Cu > Cr > Ni > As > Pb > Zn > Cd > Fe. The initial adsorption rate for all the nano-adsorbents exhibited same pattern/order for Pb, Zn, Cd and Fe, whereas for other heavy metals, the initial adsorption rate is a function of the type and properties of polymer used for the surface functionalization. For instance, it was noticed, that Cu with (*h*) value of 416.67 mg/g/min (PHB-CNTs) > 370.37 mg/g/min (P-CNTs) adsorbed rapidly more by PHB-CNTs than P-CNTs. The pseudo-second order kinetic model further suggest that the adsorption process is chemisorption which is the rate-limiting step for the adsorption of selected heavy metals from electroplating wastewater onto both nano-adsorbents. This was evident in the formation of a covalent chemical bond between the adsorbate and nano-adsorbents and tends to find sites that will maximize their coordination number with the charged surface^58^.

### Error Analysis of Isotherms

In the regression correlation coefficients (*R*^2^) from the linearization of the four isotherm models as given in Tables 5 and 6, the *R*^2^ values suggested for P-CNTs gave Temkin isotherm as a fitted model for the sorption of As, Cu, Ni, Cr and Cd than other metals; and for PHB-CNTs, Temkin isotherm model was still suitable for adsorption of Cd, As, Ni, Cr, Cu, Pb than Fe and Zn. With the bias, resulting from linearization, the data analysis were determined by two-way analysis of variance (ANOVA) without replication (P < 0.5). The statistical analysis in Table 7 gave explanation to the sorption system with respect to the four two-parameter isotherm models, and also the competition of the heavy metals for binding sites on the two nano-adsorbents. From the summary of the statistical analysis as presented in Table 7, the suitability of the isotherm models has variation less than unity for both nano-adsorbents, which implies the stability of the sorption system. It also reveal that both nano-adsorbents behaved differently in their sorption ability towards the heavy metals present in the electroplating wastewater, which gave true description of the uniqueness of each nano-adorbents as reflected in their characterization. However, the two-way ANOVA results; sum of square error (*SSE*) indicated little significant difference between the four two-parameter isotherm models used. The SSE results shows that not all four two-parameter isotherm models are suitable on their own merits in describing the potential of nano-adsorbents for the removal of heavy metals from electroplating wastewater. Nevertheless, the ANOVA data further confirmed the heavy metal sorption on the nano-adsorbents may not only be restricted to differences in the ionic/atomic radius of the heavy metals, but also on other parameters like concentration of the heavy metals in the wastewater, its hydration energy, electrostatic forces, electronegativity, and ionic mobility may also be contributing factors.

**Table 7:** Two-way analysis of variance (ANOVA) of nano-adsorbents without replication at ∝ = 0.05.

	Count	Sum	Average	Variance	SSE
**Summary (P-CNTs)**					
Langmuir	8	1.5157	0.1895	0.0331	0.0047
Freundlich	8	3.2239	0.4030	0.0817	0.0110
Temkin	8	3.8984	0.4873	0.1448	0.0204
D-R	8	3.2992	0.4124	0.0506	0.0072
**Summary (PHB-CNTs)**					
Langmuir	8	2.8356	0.3545	0.0762	0.0109
Freundlich	8	3.9732	0.4967	0.1022	0.0146
Temkin	8	4.9421	0.6178	0.1509	0.0216
D-R	8	4.0507	0.5063	0.0766	0.0110

### Thermodynamics

Thermodynamic investigation provides information on the adsorption transfer mechanism of molecules from the solution onto the solid-liquid interface under the influence of applied temperature. The results obtained for *ΔG*°, *ΔH*°, and *ΔS*° are shown in Fig. 3(j,k), and in Tables 11–14 (supplementary file). It was found that the value of *ΔG*° decreased with increasing temperature which was an evidence of a decrease in the rate of adsorption irrespective of the nano-adsorbents. The negative value of the free energy change (*ΔG*°) suggested that the adsorption process was spontaneous. While the positive value of enthalpy (*ΔH*°) implied that the adsorption process is endothermic in nature. Also, the positive value of entropy (*ΔS*°) was an evidence of increase randomness or disorderliness during the adsorption process^59^, and can be linked to accelerated dehydration steps of the adsorbate, which have been known to have relatively high energies of solvation. Moreso, an increase in the rate of adsorption (*K*_*C*_) as the temperature increases can be linked to the increase in the forces of adhesion between the nano-adsorbents and adsorbate phases^60^. The order of maximum *K*_*C*_ is as follows: PHB-CNTs > P-CNTs.

### Comparative effects of the adsorption factors of heavy metals from electroplating wastewater

The analysis showed that four factors such as contact time, dosage, pH and temperature influenced the adsorption process of the selected heavy metals (see Fig. 3(g)). Of all the investigated factors, it was found that the removal efficiency of the selected heavy metals mostly depends on the dosage of the nano-adsorbent and the nano-adsorbent exhibited similar behavioural adsorption pattern. Overall, the PHB-CNTs had the maximum removal of heavy metals compared to the P-CNTs due to enhance modification of the later. PHB-CNTs have high crystallinity, aspect ratio, surface area and porosity, increased functional group, more binding sites for adsorption amongst others. Nevertheless, studying the isotherms and kinetics of the adsorption process further demonstrated the most qualitative material in the sequestration of heavy metals from industrial electroplating wastewater. The isotherm suit best Temkin model, and kinetic fitted most to pseudo second order model. Therefore, comparing the maximum percentage removal in this study with previous researches (see Table 8), it was observed that the nano-adsorbents has outstanding capacity and capability for the treatment of electroplating wastewater. While other adsorbents required high dosage for maximum removal, the developed nano-adsorbents required less, with simultaneous and multi-sorption of the heavy metals from raw industrial electroplating wastewater. It was also noticed from the literature findings that only this study reported nano-adsorbent for heavy metals removal from industrial electroplating wastewater.

**Table 8:** Percentage removal of different adsorbent on electroplating wastewater.

Adsorbent	Dosage used (mg)	Adsorbate	Maximum Percentage removal (%)	Reference
Rice husk	367	RIEW	Cr (74), Cd (79), Cu (38), Ni (99)	^55^
Coconut coir	367	RIEW	Cr (93), Cd (40), Cu (39), Ni (99)	^55^
CAC	1000	RIEW	Cr (95)	^57^
PAC	3000	SIEW	Ni(89), Zn (90), Cr (89)	^62^
PAC-SDDC	3000	SIEW	Ni(82), Zn (85), Cr (97)	^62^
Bamboo activated carbon	200	RIEW	Ni (98)	^63^
P-CNTs	20	RIEW	Fe (92), Ni (90), Cd (99), Pb (99), Cu (93), Zn (89), Cr (99), As (100)	This study
PHB-CNTs	20	RIEW	Fe (93), Ni (90), Cd (99), Pb (99), Cu (93), Zn (86), Cr (99), As (100)	This study

Key; Raw Industrial Electroplating Wastewater (RIEW), Simulated Industrial Electroplating Wastewater (SIEW), Corncob based Activated Carbon (CAC), Powdered Activated Carbon (PAC), Powdered Activated Carbon modified with sodium diethyldithiocarbamate (PAC-SDDC), Bamboo Activated Carbon (BAC), Purified Carbon Nanotubes (P-CNTs), Polyhydroxylbutyrate functionalized Carbon Nanotubes (PHB-CNTs).

## Conclusion

In summary, the research focused on the production, functionalization and characterization of carbon nanotubes as nano-adsorbents for removal of selected heavy metals from industrial electroplating wastewater via batch adsorption process. The HRSEM/HRTEM/EDS analysis of the P-CNTs and PHB-CNTs revealed formation of a well-defined morphology with less degree of agglomeration, while the BET analysis gave surface area and porosity of the CNTs to be significantly enhanced upon functionalization with PHB. The DLS analysis showed that both nano-adsorbents are broadly polydispersed with a high aspect ratio in the range of 400–3200. XPS demonstrated the presence of functional groups (C-C, C=C, C-OH, C=O and O-C=O) as well as the presence of the π-π* bond. The batch adsorption process of heavy metals removal from electroplating wastewater is factored by contact time, dosage, temperature and pH. The heavy metals removal efficiency by the nano-adsorbents is in the order of PHB-CNTs > P-CNTs based on the high surface area and more functional groups of the former than the latter. The equilibrium sorption and kinetic data of the nano-adsorbents for the removal of heavy metals on the electroplating wastewater are better described by Temkin isotherm and pseudo-second models. The thermodynamic parameters showed that the adsorption process was spontaneous and endothermic in nature. The adsorption mechanisms involved in this study are ion exchange and electrostatic force mechanism. The novelty of this study demonstrated a one-way treatment method (adsorption) of industrial electroplating wastewater using the developed nano-adsorbents (P-CNTs and PHB-CNTs) with unique, high potential and removal capacities of heavy metals from the wastewater. In addition, the nano-adsorbents possess simultaneous multi-adsorption capacity of different heavy metal ions from raw industrial electroplating wastewater. Likewise, PHB-CNTs is a composite of biodegradable and biocompatible polymer with carbon nanotubes, this define its low toxicity, ease of production, and availability. Moreover, this is the first time PHB-CNTs was used as an adsorbent for removal of heavy metals from industrial electroplating wastewater. Overall, it was observed for the first time that adsorption behavior of both P-CNTs and PHB-CNTs are not only surface area specific but indirectly factored by their water holding capacity and attached functionality. Finally, the treated adsorbate meets the water quality standard for re-use, either for industrial or agricultural (irrigation) activities.

## Experimental

### Production of carbon nanotubes

**Figure 4:**
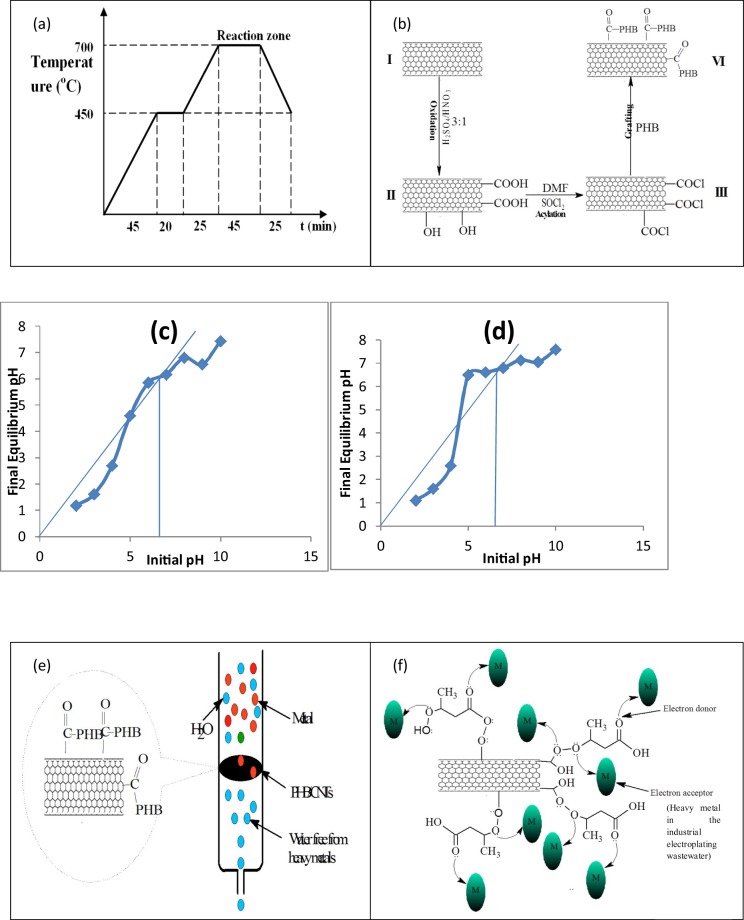
(**a**) Temperature program used in CVD for CNTs synthesis at 700 °C for 45 min. (**b**) Schematic pathway/mechanism of purified and polymer functionalized carbon nanotubes production (I = as-synthesized/un-purified carbon nanotubes, II = purified carbon nanotubes (P-CNTs), III = acyl-carbon nanotubes (acyl-CNTs), and IV = polymer functionalized carbon nanotubes (PHB-CNTs)). (**c**) Initial pH versus final equilibrium pH for P-CNTs – pH drift method for determining the zero point of discharge (pHzpc). (**d**) Initial pH versus final equilibrium pH for PHB-CNTs – pH drift method for determining the zero point of discharge (pHzpc). (**e**) The schematic diagram of removal of heavy metals from the electroplating wastewater using polymer functionalized carbon nanotubes (PHB-CNTs) via batch adsorption process. (**f**) Chemical mechanism pathway for the adsorption of heavy metals from the electroplating wastewater using polymer functionalized carbon nanotubes (PHB-CNTs) via batch adsorption process.

The procedure described by Bankole *et al*.^39^ was employed to produce CNTs. A known weight (1 g) of synthesized Fe-Co/CaCO_3_ catalyst was put in a ceramic boat and placed in a horizontal tubular quartz reactor within the furnace. The furnace was heated at rate of 10 °C/min while argon (carrier gas) was flown over the catalyst at 30 cm^3^/min for 90 min prior to the commencement of the reaction. At the reaction temperature of 700 °C, argon flow rate was increased to 230 cm^3^/min and C_2_H_2_ was introduced at flow rate of 190 cm^3^/min. The reaction proceeded for 45 min and thereafter the C_2_H_2_ flow was stopped, while flow of argon was reduced to 30 cm^3^/min for the furnace to cool down to room temperature (see Fig. 4(a)). The produced CNTs were removed from the quartz reactor and weighed to quantify the percentage yield^61^ (see Equation (1)):

1 $$ \% {\rm{CNTs}}\,=\,\frac{{w}_{3}-({w}_{1}-{w}_{2})}{{w}_{1}-{w}_{2}}\times 100$$

where *w*_1_ is the initial weight of catalyst, *w*_2_ is the weight loss of catalyst at reaction temperature and *w*_3_ is the weight of carbon deposit and catalyst.

### Purification and polymer functionalization of carbon nanotubes

The procedure described by Bankole *et al*.^39^ and Veličković *et al*.^38^ were used to purify and functionalize the CNTs respectively. The CNTs produced were purified with mixture of concentrated nitric and sulphuric acid in the ratio (v/v 1:3), to remove carbonaceous and metallic impurities present in the carbon materials. Deionized water was added to the mixture and thereafter ultrasonicated for 3 h at 40 °C. The mixture was then filtered, and the residue was thoroughly rinsed until pH of 7 was achieved. The sample was further oven-dried at 80  C for 8 h to give purified CNTs (P-CNTs) as shown Fig. 4(b). The functionalization of the P-CNTs with PHB was carried out as follow: 5 g of P-CNTs was dispersed in 10% v/v of dimethylformamide (DMF) followed by addition of 10 cm^3^ of 0.01 M Thionyl chloride and subsequently heated for 24 h, and later sieved with excess anhydrous tetrahydrofuran (THF) to form CNT-COCl. The material was then dried at 60  C for 3 h^38^. Furthermore, 15 cm^3^ of 0.1 M PHB and DMF was then added to CNT-COCl and the mixture was again heated at 35  C for 12 h. The obtained product was sonicated in 5% NaHCO_3_, centrifuged, and washed with combination of deionized water and methanol and later oven-dried at 60 °C for 3 h to give PHB-CNTs shown in Fig. 4(b). The P-CNTs and PHB-CNTs were then characterized using HRTEM, HRSEM, EDS, Nano-zetasizer, Raman, FTIR, TGA, XRD, BET and XPS.

### Heavy metal analysis of the electroplating wastewater before and after treatment with nano-adsorbents

A Flame Atomic Absorption Spectrophotometer (PG 990 model) was used to determine the concentrations of the selected heavy metals; arsenic, chromium, lead, cadmium, zinc, copper, iron and nickel in the electroplating wastewater. All these metals were analyzed using the air-acetylene, except for As in which manual gaseous hydride generation AAS method was used. Measurements were recorded with two repeatable readings before and after treatment with nano-adsorbents.

### Batch Adsorption Experiment on the Electroplating Wastewater

The removal of the selected heavy metals in the electroplating wastewater treatment by the nano-adorbents (P-CNTs and PHB-CNTs) was achieved via batch adsorption process. The percentage of adsorption (Removal%) and adsorption capacity (q_e_) of each nano-adsorbent were calculated using Equations (2) and (3).

2 $${\rm{Removal}}( \% )=\frac{{C}_{o}-\,{C}_{e}}{{C}_{o}}\times 100$$

3 $${q}_{e}=\frac{({C}_{o}-\,{C}_{e})V}{W}$$

where C_o_ is the initial concentration of the adsorbate (mg/L), C_e_ is the equilibrum concentration (mg/L), q_e_ is the amount adsorbed on unit mass of the adsorbent (mg/g), V is the volume of the adsorbate used during adsorption process (cm^3^) and W is the mass of the adsorbent used during the adsorption process (mg). In the batch adsorption process, influence of three parameters namely; contact time, adsorbent dosage, and temperature were examined. The procedure for each parameter is explained as follows.

### Effect of contact time

The influence of contact time on the removal rate of the selected heavy metals by the nano-adsorbent was investigated by adding 10 mg of the nano-adsorbents into 50 cm^3^ of the electroplating wastewater in a 250 cm^3^ conical flask. The flask was covered to create pressure and stirred continuously on rotary shaker at 190 rpm at different time interval (10, 20, 30, 40, 50, 60, 70, and 80 min). The pH of the solution was not adjusted and sampling was done periodically and filtered. The filterate was analysed for the selected heavy metals by AAS technique.

### Effect of adsorbent dosage

Different amounts of nano-adsorbents (10, 20, 30, 40, and 50 mg) were added separately to 50 cm^3^ of the electroplating wastewater in a 250 cm^3^ conical flask. The mixture was covered with rubber bunk and agitated on a rotary shaker at 190 rpm for 70 min (equilibrium time). Sampling was done periodically at every 10 minutes and and filtered. Subsequently the filterate was analysed for the selected heavy metals by AAS technique.

### Effect of temperature

A known mass **(**10 mg) of nano-adsorbent was added to 50 cm^3^ of the electroplating wastewater in a 250 cm^3^ conical flask. The flask was covered with rubber bunk and the temperature was varied as follows; 303, 313, 323, 333, and 343 K. The mixture was agitated on rotary shaker and heated to the desired temperature and at 190 rpm for 70 min (equilibrium time). The sample was filtered to remove the residue while the filtrate was analysed for the heavy metals using AAS technique.

### Effects of pH

The point zero charge (pHzpc) of the nano-adsorbents was determined by pH drift method^55^. Into a series of 100 cm^3^ beaker, 30 cm^3^ of 0.1 M KCl was added and adjustment was done on the initial pH (*pH*_*i*_) from 2–10 with 0.1 M HCl or 0.1 M KOH solutions. There was addition of 0.1 g of P-CNTs into the adjusted pH solutions, and were shaken manually and left to reach equilibrium for 48 hrs in a roto spin unit. After 48 hrs the suspensions was filtered and the final equilibrium pH (*pH*_*f*_) values of the filterate were recorded. The final equilibrium pH (*pH*_*f*_) values were plotted against initial pH (*pH*_*i*_) values. Similarly, same experiments were carried out to determine the point zero charge (pHzpc) of PHB-CNTs. The plots are presented in Fig. 4(c,d).

### Chemical pathway and adsorption mechanism of the batch adsorption process

The diagram of batch adsorption process of the electroplating wastewater using PHB-CNTs was demonstrated via chemical pathways shown in the schematics (see Fig. 4(e,f)). In Fig. 4(e), it shows the electroplating wastewater passing through PHB-CNTs, filtered out as water clean from heavy metals. The nano-adsorbents are novel materials which contain different functional groups on the external surface, and are enhancer to the adsorption process. Figure 4(f) shows a chemical mechanism pathway between PHB-CNTs and electroplating wastewater, where the heavy metal ions (M) in the electroplating wastewater attached to the functional groups on the surface of PHB-CNTs (electron donor and binding sites). Due to surface modification of CNTs with PHB, there was increase in the surface positive charges (confirmed by the pH_zpc_ of PHB-CNTs) as a result of protonation of the carboxyl and carbonyl groups in aqueous solution^22^. There was transfer of the ligand charge of functional groups to the heavy metal ions in the wastewater. The van der Waal forces within the nano-adsorbent breaks and there was electrons transfer from the nano-adsorbent to form a coordinate-covalent bond with the anion of the heavy metals. These create sorbate/sorbent relationship that resulted to removal of the selected heavy metal ions from the electroplating wastewater.

### Langmuir isotherm

The Langmuir isotherm was used to determine the maximum adsorption capacity produced from complete monolayer coverage of adsorbent surface as shown in the isotherm Equation (4)

4 $${q}_{e}=\frac{{a}_{L}{C}_{e}}{1+{b}_{L}{C}_{e}}$$

which on linearization of Equation (4) becomes Equation (5)

5 $$\frac{1}{{q}_{e}}=\frac{1}{{a}_{L}}.\frac{1}{{C}_{e}}+\frac{{b}_{L}}{{a}_{L}}$$

where q_e_ depicts the concentration of metal ions in solution at equilibrium after sorption, C_e_ represents the concentration of liquid at equilibrium with that of the solid phase, *a*_*L*_ and *b*_*L*_ are empirical constants of the Langmuir model, *b*_*L*_ is the adsorption equilibrium constant (dm^3^/mg) related to the apparent energy of adsorption and *Q*_*o*_ is the maximum monolayer coverage capacities (mg/g). A plot of *1/q*_*e*_ against *1/C*_*e*_ will give straight line with 1/a_L_ as the slope and b_L_/a_L_ the intercept, while *Q*_*o*_ was obtained from *a*_*L*_*/b*_*L*._ The separation factor, *R*_L_ was obtained using Equation (6):

6 $${R}_{L}=\frac{1}{1+{b}_{L}{C}_{e}}$$

The value of *R*_*L*_ lies between 0 and 1 for a favorable adsorption. An *R*_*L*_ > 1, it represents an unfavorable adsorption, while *R*_*L*_ = 1 shows the linear adsorption, while the adsorption process is irreversible if *R*_*L*_ = 0.

### Freundlich isotherm

The Freundlich isotherm can be expressed as shown in Equation (7):

7 $${q}_{{\rm{e}}}={K}_{f}\,{C}_{e}^{n}$$

where *K*_f_ is the Freundlich constant which gives the relative adsorption capacity of the adsorbent related to the bonding energy, and *n*_*F*_ is the heterogeneity factor or Freundlich coefficient. On linearization of Equation (7), the plot of log *q*_e_ against log *C*_e_ was used to determine Freundlich coefficient as given in Equation (8).

8 $$Log\,{q}_{e}=Log\,{K}_{f}+{n}_{F}Log\,{C}_{e}$$

### Temkin isotherm

The nonlinear form of Temkin equation is shown in Equation (9).

9 $${q}_{e}=\frac{RT}{{b}_{T}}In\,({A}_{T}{C}_{e})$$

And when Equation (9) is linearized, it gave Equation (10)

10 $${q}_{e}={B}_{T}\,In\,{A}_{T}+{B}_{T}\,In\,{C}_{e}$$

where, B_T_ = (RT)/b_T_, T is the absolute temperature (K) and *R* stands for universal gas constant, 8.314 J mol^−1^ K^−1^. *A*_*T*_ is the equilibrium binding constant/maximum binding energy (L/g), while *b*_*T*_ is related to the heat of adsorption. A plot of *qe* versus *ln C*_*e*_ determines the isotherm constants *A*_*T*_ (slope) and *b*_*T*_ (intercept).

### Dubinin-Radushkevich (D-R)

D-R model is an isotherm model, which is application of the mechanism of adsorption with a Gaussian energy distribution on a heterogeneous surface. It does not assume expression on homogeneous surface or constant adsorption potential. The D-R equation is given in Equation (11) and on linearization it gave Equation (12)

11 $${q}_{e}={q}_{s}\exp (-{K}_{ads}\varepsilon )$$

12 $$In{q}_{e}=In\,{q}_{s}-{K}_{ads}{\varepsilon }^{2}$$

*q*_*s*_ (mg/g) is the theoretically capacity of saturation, *K* is known as *K*_*ads*_*/D–R* constant (mean free energy of adsorption) (mol^2^/kJ^2^), and *ε* is the Polanyi potential and it is calculated using Equation (13)

13 $$\varepsilon =RT\,In[1+\frac{1}{{C}_{e}}]$$

The values of *q*_*s*_ and *K* were determined by plotting *ln q*_*e*_ against *ε*^2^. The mean adsorption energy (*E*) (kJ/mol) can be calculated using Equation (14)

14 $$E=[\frac{1}{\sqrt{2{K}_{ads}}}]$$

when the value of *E* < 8 KJ/mol, the adsorption process is physiosorption, while for value of *E* between 8 and 16 kJ/mol, the process is of chemisorptions or by ion-exchange.

### Pseudo first-order kinetics

The non-linear form of pseudo first-order equation is given by Equation (15):

15 $$\frac{d{q}_{t}}{{d}_{t}}={k}_{ad}\,({q}_{e}-{q}_{t})$$

where, *q*_*e*_ and *q*_*t*_ are quantity of metal ion sequestered (mg/g) at equilibrium time and at any instant of time, *t* respectively, and *k*_*ad*_ (min^−1^) is the rate constant of the pseudo first order adsorption. The integrated rate law after application of the initial condition of *q*_*t*_ = 0 at *t* = 0, becomes a linear equation shown in Equation (16):

16 $$Log({q}_{e}-{q}_{t})=Log\,{q}_{e}-{k}_{ad}\,t/2.303$$

Plot of *log (q*_*e*_ − *q*_*t*_) verus *t* gives a straight line for the pseudo first order adsorption kinetics.

### Pseudo second-order kinetic model

The rate equation used to test the applicability of the second order kinetics is given in equation (17)

17 $$\frac{d{q}_{t}}{{d}_{t}}={k}_{2}{({q}_{e}-{q}_{t})}^{2}$$

where, *k*_2_ (g mg^−1^ min^−1^) is the second order rate constant. From the boundary conditions, *t* = 0 to *t* = *t* and *q*_*t*_ = 0 to *q*_*t*_ = *q*_*t*_, the integrated form of the equation becomes as shown in Equation (18):

18 $$\frac{1}{({q}_{e}-{q}_{t})}=\frac{1}{{q}_{e}}{k}_{2}t$$

when Equation (18) is linearizing, it is given by Equation (19):

19 $$\frac{t}{{q}_{t}}=\frac{1}{h}+(\frac{1}{{q}_{e}})t$$

As the initial sorption rate with *t* → 0, *h* = *k*_2_*q*_*e*_^2^. On this condition, the plot of *t/q*_*t*_ versus *t* should give a linear relationship, which further aids the computation of *q*_*e*_, *k*_2_ and *h*.

### Elovich kinetic model

Elovich Kinetic Equation is a rate equation based on the adsorption capacity which is expressed Equation (20):

20 $$\frac{d{q}_{t}}{{d}_{t}}=\alpha \,\exp (-\,\beta {q}_{t})$$

where ***α*** is the initial rate of adsorption (mg g^−1^ min^−1^) and ***β*** is desorption constant/extent of the surface coverage/activation energy for chemisorption (g/mg). On simplification of Equation (3.35), assuming ***αβ*** ≫ *t* and by application of the boundary conditions *q*_*t*_ = 0 at *t* = 0 and *q*_*t*_ = *q*_*t*_ at *t* = *t*, as shown in Equation (21):

21 $${q}_{t}=\frac{1}{\beta }In(\alpha \beta )+\frac{1}{\beta }In\,t$$

The slope and intercept of the plot of *q*_*t*_ versus *ln t* determines the kinetic constants, ***α*** and ***β***.

### Fractional power kinetic model

This kinetic model is a modified form of the Freundlich equation and is expressed in Equation (22)

22 $$Log\,{q}_{t}=Log\,{K}_{3}+v\,Log\,t$$

where, *K*_3_ and *ν* are constants with *ν* < 1. The functions *K*_3_ and *ν* are constants, being the specific sorption rate at unit time, when *t* = 1. The plot of *Log q*_*t*_ against Log *t* will show a linear relationship while value of *K*_3_ and *ν* can be obtained from the intercepts and slopes of the plots respectively.

### Thermodynamics

The effect of temperature aids in the evaluation of the change in free energy (*ΔG°*), enthalpy (*ΔH°*) and entropy (*ΔS°*). The change in free energy was estimated using the relation in Equation (23)

23 $$\Delta {G}^{o}=-\,RTln{K}_{c}$$

*T* (K) denotes the absolute temperature; *R* is the gas constant (kJ/mol). The equilibrium constant (*K*_*c*_) was evaluated using Equation (24):

24 $${K}_{c}={C}_{ad}/Ce$$

where *C*_*e*_ and *C*_*ad*_ are the equilibrium concentrations of metal ions (mg/L) in solution and on adsorbent respectively. Enthalpy and entropy were obtained using Van’t Hoff equation (see Equation 25)

25 $${\rm{\Delta }}{G}^{^\circ }={\rm{\Delta }}{H}^{^\circ }-T{\rm{\Delta }}{S}^{^\circ }$$

On substituting Equation (25) into Equation (23), it gives Equation (26)

26 $$Log\,{K}_{C}=\frac{{\rm{\Delta }}{G}^{^\circ }}{RT}=\frac{{\rm{\Delta }}{S}^{^\circ }}{2.303R}-\frac{{\rm{\Delta }}{H}^{^\circ }}{2.303R}\{\frac{1}{T}\}$$

Thermodynamic parameters such as the change in Gibbs free energy *ΔG*^0^ (kJ/mol) were estimated using the classical Vant Hoff equation at different temperatures. *ΔH*^0^ (kJ/mol) is the change in enthalpy and *ΔS*^0^ (kJ/mol/K) is the change in entropy. Therefore *ΔH*^0^ and *ΔS*^0^ were determined by the slope and intercept of linear Vant Hoff’s plot (Log *K*_*C*_ versus (*1/T*)) respectively.

## Supplementary information

Supplementary Document

## Data Availability

All data generated or analysed during this study are included in this published article (and its Supplementary Information files).
