# Supplementary material for: Selected Heavy Metals Removal From Electroplating Wastewater by Purified and Polyhydroxylbutyrate Functionalized Carbon Nanotubes Adsorbents
Source: Sci Rep. 2019 Mar 14;9:4475. doi: 10.1038/s41598-018-37899-4 (PMC6418241; doi:10.1038/s41598-018-37899-4)
Supplement: Supplementary file 1 — Supplementary Document [file 41598_2018_37899_MOESM1_ESM.pdf]

SELECTED HEAVY METALS REMOVAL FROM ELECTROPLATING WASTEWATER BY PURIFIED AND POLYHYDROXYLBUTYRATE FUNCTIONALIZED CARBON NANOTUBES ADSORBENTS

Mercy Temitope Bankole[1,5*], Ambali Saka Abdulkareem[2,5], Ishaq Alhassan Mohammed[2,5], Stephen Shaibu Ochigbo[1], Jimoh Oladejo Tijani[1,5], Oladiran Kamaldeen Abubakre[3,5], Wiets Daniel Roos[4]

[1]Department of Chemistry, Federal University of Technology, PMB.65, Minna, Niger State, Nigeria

[2]Department of Chemical Engineering, Federal University of Technology, PMB.65, Minna, Niger State, Nigeria

[3]Department of Mechanical Engineering, Federal University of Technology, PMB.65, Minna, Niger State, Nigeria

[4]Department of Physics, University of the Free State, P.O. Box 339, ZA-9300 Bloemfontein, Republic of South Africa

[5]Nanotechnology Research Group, Centre for Genetic Engineering and Biotechnology (CGEB), Federal University of Technology, P.M.B 65, Bosso, Minna, Niger State, Nigeria

***Corresponding Authour E-mail: bankole.temitope@futminna.edu.ng, Phone Number: +2348055452572**

Supplementary Information

Table 9: Kinetic Parameters for the Adsorption of Heavy Metals on P-CNTs

**Kinetic models**	**Fe**	**Ni**	**Cd**	**Pb**	**Cu**	**Zn**	**Cr**	**As**
**Pseudo-first order**								
K_1 (/min)	-0.045	-0.041	0.042	-0.016	-0.036	-0.069	-0.056	-0.059
q_e (mg/g)	58.18	16.54	0.03	0.43	15.84	60.26	71.95	24.11
R^2	0.6615	0.3052	0.5630	0.1112	0.4003	0.4866	0.8366	0.326
**Pseudo-second order**								
K_2(g/mg/min	0.0011	0.0019	0.2022	0.1177	0.0021	0.0019	0.0015	0.0040
q_e (mg/g)	107.53	416.67	14.93	24.63	416.67	185.19	370.37	294.12
h (mg/g/min)	12.854	322.581	45.045	71.429	370.370	64.103	204.082	344.828
R^2	0.9563	0.9979	0.9999	0.9999	0.9985	0.9909	0.9994	0.9999
**Elovich**								
β (g min/mg)	0.118	0.277	70.423	6.882	0.280	0.218	0.077	0.168
α(g min^2/mg)	7832.7	6×10^{47}	-	3×10^{70}	2×10^{47}	3×10^{15}	2×10^{11}	9×10^{19}
R^2	0.322	0.0427	0.0086	0.4078	0.0598	0.1045	0.8014	0.8837
**Fractional power**								
K_3 (mg/g)	62.79	392.83	14.75	28.85	384.41	157.65	305.42	264.67
ν (min^{-1})	0.0927	0.0089	0.0010	0.0060	0.0090	0.0260	0.0373	0.0210
K_3ν(mg/g/min	5.8208	3.4962	0.0148	0.1431	3.4597	4.0990	11.3922	5.5581
R^2	0.2945	0.0412	0.0085	0.4074	0.0578	0.0974	0.8046	0.8849

Table 10: Kinetic Parameters for the Adsorption of Heavy Metals on PHB-CNTs

Kinetic models	Fe	Ni	Cd	Pb	Cu	Zn	Cr	As
**Pseudo-first order**								
K_1 (/min)	-0.04	-0.05	-0.07	0.03	-0.03	-0.05	-0.06	-0.07
q_e (mg/g)	30.76	88.39	665.58	0.06	10.05	43.03	76.97	66.74
R^2	0.6072	0.5673	0.5716	0.3138	0.2205	0.4494	0.9186	0.6066
**Pseudo-second order**								
K_2 (g/mg/min	0.0017	0.0009	0.1271	0.1351	0.0024	0.0012	0.0015	0.0018
q_e (mg/g)	106.38	434.78	14.06	24.33	416.67	161.29	357.14	294.12
h (mg/g/min)	18.832	175.43	28.818	80.000	416.67	31.447	188.68	153.85
R^2	0.9774	0.9953	0.9999	0.9999	0.9986	0.9790	0.9996	0.9997
**Elovich**								
β (g min/mg)	0.20	0.11	9.74	16.78	0.3863	0.1225	0.0649	0.0733
α(gmin^2/mg)	2×10^{7}	6×10^{18}	2×10^{60}	9×10^{172}	6×10^{65}	7.4×10^{6}	$2.\times10^{9}$	3.1×10^{8}
R^2	0.1858	0.1520	0.3378	0.0658	0.0359	0.2195	0.8982	0.9426
**Fractional power**								
K_3 (mg/g)	76.24	376.35	14.481	23.9056	389.58	115.03	291.41	234.64
ν (min^{-1})	0.0525	0.0227	0.0069	0.0025	0.0065	0.0565	0.0451	0.0491
K_3ν(mg/g/min	4.0028	8.5433	0.0999	0.0598	2.5323	6.4990	13.1425	11.5208
R^2	0.1671	0.1443	0.3359	0.0652	0.0345	0.2009	0.9012	0.9457

Table 11: Thermodynamic Parameters for Adsorption of Heavy Metals on P-CNTs

Temperature (◦C)	ΔG^0 (kJ/mol)	ΔS^0 (kJ/ mol/ K)	ΔH^0 (kJ/mol)	K_C
**Fe**		0.2453	4813.64	
30	-4887.95			0.1437
40	-4890.41			0.1527
50	-4892.86			0.1617
60	-4895.31			0.1706
70	-4897.76			0.1795
**Ni**		5.7832	4419.39	
30	-6171.71			0.0863
40	-6229.54			0.0913
50	-6287.37			0.0962
60	-6345.20			0.1011
70	-6403.03			0.1059
**Cd**		18.0979	6630.08	
30	-12113.8			0.0082
40	-12294.7			0.0089
50	-12475.7			0.0096
60	-12656.7			0.0103
70	-12837.7			0.0111
**Pb**		195.2127	63463.30	
30	-122613			7.28×10^{-22}
40	-124565			1.63×10^{-21}
50	-126517			3.46×10^{-21}
60	-128469			7.04×10^{-21}
70	-130421			1.37×10^{-20}

Table 12: Thermodynamic Parameters for Adsorption of Heavy Metals on P-CNTs

Temperature (◦C)	ΔG^0 (kJ/mol)	ΔS^0 (kJ/ mol/ K)	ΔH^0 (kJ/mol)	K_C
**Cu**		4.6326	3897.52	
30	-5301.19			0.1219
40	-5347.51			0.1281
50	-5393.84			0.1342
60	-5440.16			0.1402
70	-5486.49			0.1460
**Zn**		1.5356	3662.90	
30	-4128.18			0.1942
40	-4143.53			0.2035
50	-4158.90			0.2125
60	-4174.25			0.2214
70	-4189.61			0.2301
**Cr**		40.1516	8191.37	
30	-20357.3			0.0309×10^{-2}
40	-20758.8			0.0343×10^{-2}
50	-21160.3			0.0378×10^{-2}
60	-21561.9			0.0415×10^{-2}
70	-21963.4			0.0452×10^{-2}
**As**		88.3612	14358.30	
30	-41131.7			8.11×10^{-8}
40	-42015.3			9.73×10^{-8}
50	-42898.9			1.15×10^{-7}
60	-43782.6			1.36×10^{-7}
70	-44666.2			1.58×10^{-7}

Table 13: Thermodynamic Parameters for Adsorption of Heavy Metals on PHB-CNTs

Temperature (∘C)	ΔG^0 (kJ/mol)	ΔS^0 (kJ/ mol/ K)	ΔH^0 (kJ/mol)	K_C
**Fe**		-0.0025	4811.06	
30	-4810.31			0.1482
40	-4810.28			0.1575
50	-4810.26			0.1668
60	-4810.23			0.1760
70	-4810.21			0.1851
**Ni**		-4.3690	809.73	
30	514.08			1.2264
40	557.77			1.2391
50	601.46			1.2510
60	645.15			1.2324
70	688.84			1.2732
**Cd**		9.8554	2403.41	
30	-5389.60			0.1177
40	-5488.16			0.1214
50	-5586.71			0.1249
60	-5685.26			0.1283
70	-5783.82			0.1316
**Pb**		20.4740	7125.85	
30	-13329.5			0.0050
40	-13534.2			0.0055
50	-13739.0			0.0060
60	-13943.7			0.0065
70	-14148.4			0.0070

Table 14: Thermodynamic Parameters for Adsorption of Heavy Metals on PHB-CNTs

Temperature (◦C)	ΔG^0 (kJ/mol)	ΔS^0 (kJ/ mol/ K)	ΔH^0 (kJ/mol)	K_C
**Cu**		1.0052	2406.9	
30	-2711.47			0.3408
40	-2721.52			0.3514
50	-2731.57			0.3616
60	-2741.62			0.3715
70	-2751.67			0.3810
**Zn**		1.3278	3499.11	
30	-3901.42			0.3125
40	-3914.70			0.2222
50	-3927.98			0.2316
60	-3941.25			0.2409
70	-3954.53			0.2499
**Cr**		10.9338	4302.91	
30	-7615.83			0.0487
40	-7725.17			0.0514
50	-7834.51			0.0541
60	-7943.85			0.0567
70	-8053.18			0.0594
**As**		39.9463	5747.3	
30	-17851.0			8.37×10^{-4}
40	-18250.5			9.00×10^{-4}
50	-18649.9			9.64×10^{-4}
60	-19049.4			10.28×10^{-4}
70	-19448.9			10.92×10^{-4}
